# Ubiquitin–proteasome system and the role of its inhibitors in cancer therapy

**DOI:** 10.1098/rsob.200390

**Published:** 2021-04-28

**Authors:** Fatemeh Aliabadi, Beheshteh Sohrabi, Ebrahim Mostafavi, Hamidreza Pazoki-Toroudi, Thomas J. Webster

**Affiliations:** ^1^ Physiology Research Center, Faculty of Medicine, Iran University of Medical Sciences, Tehran, Iran; ^2^ Department of Chemistry, Surface Chemistry Research Laboratory, Iran University of Science and Technology, PO Box 16846-13114, Tehran, Iran; ^3^ Department of Chemical Engineering, Northeastern University, Boston, MA 02115, USA; ^4^ Stanford Cardiovascular Institute, Stanford, CA, USA; ^5^ Department of Medicine, Stanford University School of Medicine, Stanford, CA, USA; ^6^ Department of Physiology, Faculty of Medicine, Iran University of Medical Sciences, Tehran, Iran

**Keywords:** cancer, targeted therapy, ubiquitin–proteasome system, ubiquitination inhibitors, protein degradation inhibitors

## Abstract

Despite all the other cells that have the potential to prevent cancer development and metastasis through tumour suppressor proteins, cancer cells can upregulate the ubiquitin–proteasome system (UPS) by which they can degrade tumour suppressor proteins and avoid apoptosis. This system plays an extensive role in cell regulation organized in two steps. Each step has an important role in controlling cancer. This demonstrates the importance of understanding UPS inhibitors and improving these inhibitors to foster a new hope in cancer therapy. UPS inhibitors, as less invasive chemotherapy drugs, are increasingly used to alleviate symptoms of various cancers in malignant states. Despite their success in reducing the development of cancer with the lowest side effects, thus far, an appropriate inhibitor that can effectively inactivate this system with the least drug resistance has not yet been fully investigated. A fundamental understanding of the system is necessary to fully elucidate its role in causing/controlling cancer. In this review, we first comprehensively investigate this system, and then each step containing ubiquitination and protein degradation as well as their inhibitors are discussed. Ultimately, its advantages and disadvantages and some perspectives for improving the efficiency of these inhibitors are discussed.

## Graphical abstract

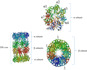

## 1. Introduction

Cells can destroy old organelles and also misfolded proteins in two ways: autophagy and the ubiquitin–proteasome system (UPS). In the 1980s, for the first time, ubiquitin and regulated destroying of proteins were investigated by Hershko and Varshavsky [1–4]. Lately, to understand ubiquitin and its related proteolysis, Hershko *et al*. discussed the E_1_, E_2_ and E_3_ enzymes more. These developments were followed by other discoveries in Hershko's laboratory. These discoveries explained the UPS biology and revealed its necessity for protein destruction, its special physiological function (in cell cycles, DNA repair, protein synthesis, transcription and stress response), its selected source (short-term signals in the special destruction of proteins) and its key mechanical features such as polyubiquitinated chain and subunit selectivity for protein destruction. Indeed, these findings resulted in a major expansion in the ubiquitin field during the 1990s. Additionally, following the discovery of proteolysis through the UPS by Hershko *et al*. [3], the biological discoveries of Varshavsky [1] in 1980 led to a basic understanding of circuits inside cells. These studies led to Hershko, Rose and Ciechanover receiving the Noble Prize in Chemistry in 2004.

Proteasomes are in all eukaryotes, bacteria, and archaea; in eukaryotes they are in either the nucleus or cytoplasm. Generally, their function is to destroy misfolded and oxidized proteins. Misproduced proteins are often found in mitochondria. The mitochondrion is an unusual organ surrounded by two membranes that protect a cell's genome. The mitochondrial genome is so small but has many varieties, which result in divergent evolutionism. The mitochondrion, as a metabolic organ, has its own DNA (mtDNA), which is maternally inherited [5]. There is some research showing that mtDNA could be independent of the nuclear DNA [6]. Mitochondrial defects have an important role in cancer development. In most research, it is noted that mtDNA mutations occur mainly on the D loop, which is a control site for expression in the mitochondrial genome. Furthermore, mutations in the control region of the D loop cause decreased expression of NADH (nicotinamide adenine dinucleotide (NAD) + hydrogen (H)) and ND6 (NADH–ubiquinone oxidoreductase chain 6 protein) [7]. Interestingly, the combination of mtDNA proteins forms a nucleoid complex with less resistance against mutations [8]. Therefore, mutations in mtDNA are greater in number than those in nuclear DNA [9]. These mutations are due to the production of reactive oxygen species (ROS) by the phosphorylation process. The high sensitivity of mtDNA to mutations is caused by ROS and the lack of protecting histones, which are the Achilles' heels of this organ, resulting in an inefficient repairing system. Fortunately, the mitochondrial network is too active to be exactly regulated in stressful conditions. These mutations play a critical role in the ageing of the tissues and cells such as the brain, muscles and fibroblasts and lots of pathological conditions like neural, metabolical and age-dependent disorders [9–11], and proteasomes are so important to protect the body against these events [12].

It has been several decades since the discovery of UPS by Ciechanover, Hershko and Rose. Nowadays, of course, new inhibitors such as new classes of anti-cancer drugs that target UPS present efficient therapeutic tools [13]. In this review, we have gathered together all inhibitors of this system to produce a universal source for new research, which is in process. Finally, a future perspective about improving these inhibitors is presented.

## 2. Proteasome structure

Proteasomes are small complexes of proteins, which are similar in size to ribosomes. Components of proteasomes are usually named by the Svedberg coefficient (S marker). The cytosolic 26S proteasome is a proteasome with a 2000 kDa weight and is found only in mammals. Furthermore, it has one 20S core subunit and two 19S regulatory caps (RP) complexes in molecular weight. Its core is empty and is therefore suitable for destroying proteins. Moreover, its openings, which are at the end of the core, allow them to come to the core. These openings are in contact with 19S caps, which are regulatory subunits having several ATPase active sites. These caps deubiquitinate proteins and admit them to the core particle (CP) [14]. There is another subunit that acts like the 19S cap called the 11S complex, which is in relation to the CP and expressed in immune responses. The 11S complex usually plays a role in destroying proteins produced in viral infections.

### 2.1. 20S subunit

**Figure 1.**
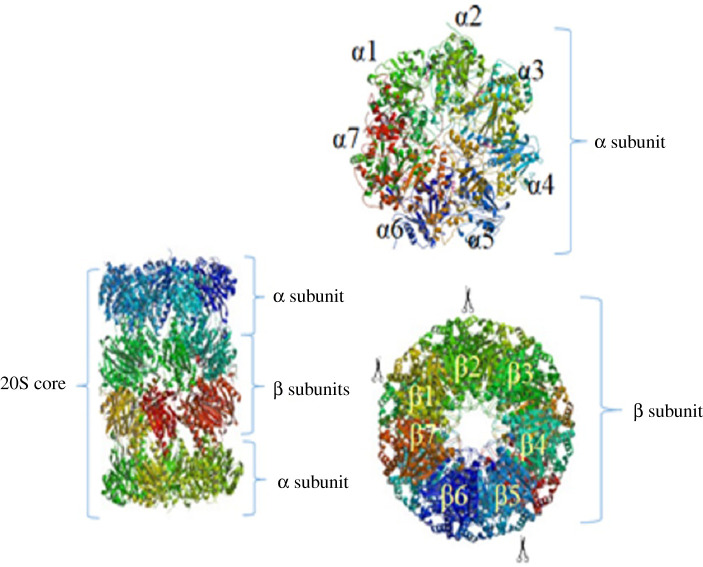
The 20S CP structure and its subunits. 20S complexes have four heptameric rings with two separate subunits: α and β subunits. α subunits are located in two ends of the proteasome core. Two inner rings are formed from seven β subunits in which β1, β2 and β3 act as caspase, trypsin and chymotrypsin, respectively.

The variety and the number of 20S subunits are related to the organism. The multi-cellular living cells contain more professional subunits than mono-cellular ones. All 20S complexes have four heptameric rings that each have two separate α and β subunits. α subunits are structural subunits, while β subunits are catalytic (figure 1). Remarkably, these subunits are pseudo-enzymes and also are homologous with each other. These subunits are adjacent by their N-terminal domains [15]. Generally, α subunits are located in two ends of the CP whose N-terminal domains (Pfam PF 10 584) form a gate not allowing unwanted proteins to come to the CP [16]. Two inner rings are formed from seven β subunits having protease activity, which proteolyse the proteins. Notably, β_1_, β_2_ and β_3_ act as caspase, trypsin and chymotrypsin, respectively [17]. The alternative forms of these subunits are β_1i_, β_2i_ and β_5i_, which are produced in response to the invasive signals such as cytokines, especially γ-interferon (IFN-γ). The proteasomes having these subunits are called immunoproteasomes [18].

### 2.2. 19S regulatory cap

**Figure 2.**
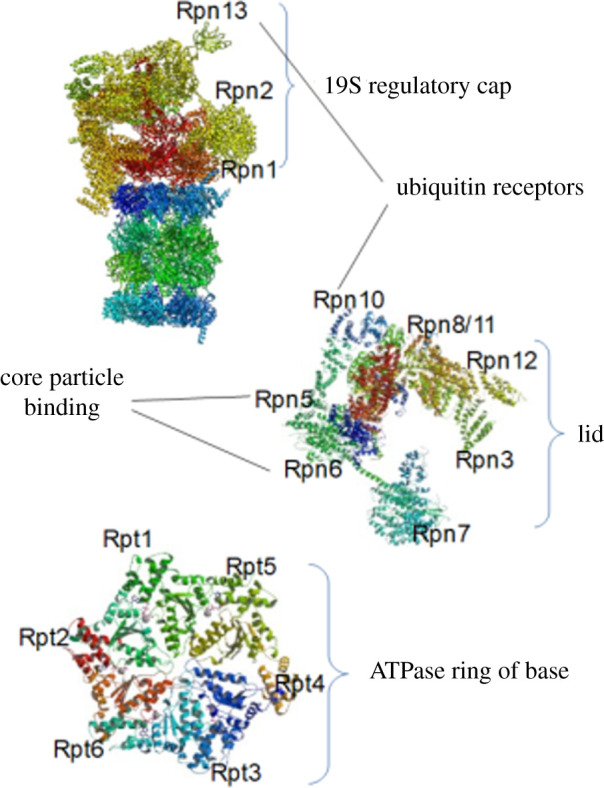
Structure of the 19S regulatory cap. The 19S regulatory cap is divided into two subunits: a base with nine proteins (Rpt1–6, Rpn1, 2, 13) in which six of them are ATPase and directly connected to the α subunit, and a lid with 10 proteins (Rpn3, 5–12, 15).

In eukaryotes, the RP has 19 proteins and also is divided into two subunits: a base with nine proteins (Rpt1–6, Rpn1, 2, 13), six of which are adenosine triphosphate hydrolyse (ATPase) directly connected to the α subunit and a lid with 10 proteins (Rpn3,5–12,15) (figure 2) [19].

Among these proteins, Rpn2 is a great target for proteasome inhibition because mutation in this protein can block the proteasome (electronic supplementary material, S1). As to the vital role of Rpt2 ATPase in regulating the CP, a mutation can cause a failure in CP channel opening. Notably, this mutation can lead to three events: (i) it can modify the active sites of CP, (ii) it can close an assumptive channel within the centre of the RP base, and (iii) any obstacle interrupts the substrate passage, but the CP channel itself is closed.

Interestingly, in the UPS protein degradation process, the only step requiring ATP hydrolysis is the step related to protein unfolding [20,21]. In fact, ATP binding to the ATPase could support the other steps and does not require ATP hydrolysis. Notably, this could lead one to achieve new types of inhibitors that indirectly inhibit proteasomes (electronic supplementary material, S1). The base subunit of the 19S cap acts as a gate and imports the proteins, while the lid acts as a deubiquitinase. In fact, the 19S cap stimulates the 20S core to degrade the proteins. The primary function of the 19S cap is opening the gate and letting the proteins come to the CP [22]. The mechanism by which the proteasome opens the gate has recently been defined [16]. These mechanisms are summarized in several steps; the C-terminal of the ATPase binds to a box in the topmost part of 20S, and consequently, the unfolded protein comes to the CP to be destroyed. The binding of the C-terminal domain of the ATPase to this box stimulates the opening of the proteasome gate. This mechanism is similar to the mechanism of a lock and key [16].

### 2.3. 11S regulatory complex

20S subunits can also bind with another regulatory complex named 11S. 11S is a heptameric structure that does not have any ATPase and has three α subunits and three β subunits. What is important is that it can degrade only small peptides, and it cannot degrade a complete protein due to its inability to unfold long proteins. This complex is also known as REG, PA28 (proteasome activator 28) and PA26 (proteasome activator 26) [15]. To open the gate of the proteasome, the 11S complex acts like the 19S cap [23]. Generally, the expression of the 11S complex relates to the immunoproteasome production in response to IFN-γ expression to produce peptides connected to the major histocompatibility I (MHC I) in viral infections.

## 3. Assembly of proteasomes

To understand how the protein is destroyed, it is necessary to study how proteasome components are assembled. Several chaperones play a role in the efficient assembly of the 20S proteasome. For instance, to study the starting point and the development of the proteasome's assembly, chaperones simultaneously contact precursors. However, there is little information about the 19S regulatory cap's assembly [24].

## 4. Ubiquitination process

**Figure 3.**
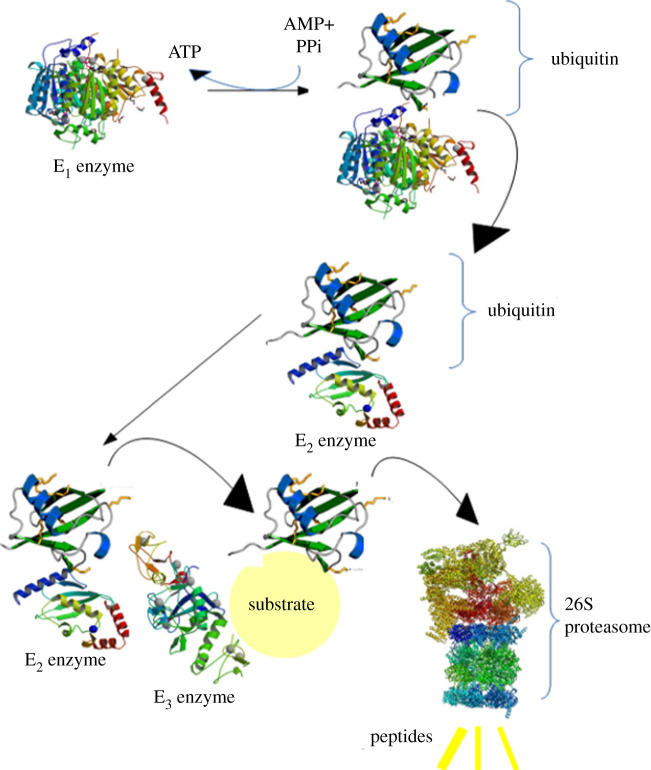
Ubiquitination process. First, E_1_ hydrolyses ATP and then adenylates one ubiquitin molecule. Later, this ubiquitin is translocated to the cysteine active site of E_1_. After that, this adenylated ubiquitin is translocated to the cysteine of E_2_. Finally, E_3_ recognizes the targeted protein and catalyses the translocation of the ubiquitin from E_2_ to the protein.

The first step of the protein degradation process is protein ubiquitination. Proteasomes target proteins for degradation that require three enzymes. As figure 3 illustrates, first, a ubiquitin-activating enzyme (E_1_) hydrolyses the ATP and then adenylates one ubiquitin molecule. Later, this ubiquitin is translocated to the cysteine active site of E1 [25]. Finally, adenylated ubiquitin is translocated to the second enzyme, cysteine, the ubiquitin conjugated enzyme (E_2_). An enzyme called ubiquitin ligase (E_3_) recognizes the targeted protein and catalyses the translocation of the ubiquitin from E_2_ to the protein. What is significant is that the targeted protein should be marked by at least four ubiquitin molecules and this must happen before recognition of the proteins by the lid [26]. Moreover, the ubiquitin molecules are conjugated to each other by the leucine residue and form a ubiquitin chain. After ubiquitination, the ubiquitin receptor should recognize the targeted protein. These receptors have one ubiquitin-like N-terminal domain (UBL) and one or more ubiquitin connected ridge(s) (UBA). RP regulates UBL, and UBA is conjugated to the ubiquitin by three α-helix bonds. These receptors may accompany the polyubiquitinated proteins to the proteasome [27].

## 5. Protein degradation process

As we previously discussed, the protein is identified by RP after being ubiquitinated. This protein should then come to CP contact the proteases [14,21]. First, it should be deubiquitinated and this deubiquitination leads to the formation of a nuclear proteolytic compartment that stimulates the proteasome's catalytic activity [28]. The hole inside the CP is too narrow (almost 13 Å) and is filled by the N-terminal domains of α subunits; consequently, at first, the proteins should be unfolded [14]. It is noteworthy that several studies have been completed based on creating a mutation in the α subunit to inhibit proteasome activity (electronic supplementary material, S1). The passage of the proteins from CP is called translocation [14,21]. Remarkably, to translocate from the CP, all proteins should be unfolded up to 20 amino acids [14]. One of the most important things is that some characters can inhibit the process of degradation. For instance, disulfide bonds are inhibitors for this process [29]. Moreover, long sequences of glycine and alanine may inhibit the unfolding process, decreasing protein degradation quality [30]. Interestingly, mutations that interrupt this process can help in the discovery of an efficient way to inhibit the proteasome (electronic supplementary material, S1). Generally, the degradation process results in the formation of small peptides, which can be degraded to smaller amino acid chains by other processes [31].

## 6. Targeting the ubiquitin–proteasome system for cancer therapy

**Figure 4.**
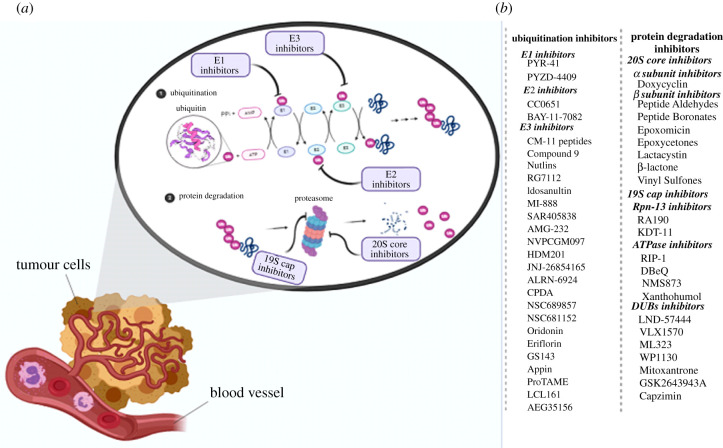
(*a*) Ubiquitin-proteasome system inhibitors. (*b*) List of the inhibitors of the ubiquitination process as well as protein degradation.

Although there is no efficient algorithm for treating cancer in a relapsed condition, using some general principles could be beneficial. For patients in a relapsed condition in the first period of their disease, using single agents depending on what was used for their initial treatment and treatment-related toxicity is a reasonable approach. Furthermore, for those patients who have not had grafts as part of their treatment or have benefited from grafts for a long duration, an autograft is the best choice. Moreover, for patients with development-relapsed conditions or invasive diseases, using new agents in combination with cytotoxic agents may be more appropriate [32]. A group of agents used in these conditions include those inhibiting the UPS. The clinical success of Bortezomib for the treatment of multiple myeloma (MM) illustrated that targeting the UPS is valid and possible [33]. What is notable is that we could target two main processes in this system: ubiquitination and protein degradation. To inhibit ubiquitination, we can target three parts, including E_1_, E_2_ and E_3_ enzymes. We can also block protein degradation, which includes inhibition of ubiquitin recognition, ubiquitin separation, protein unfolding and protein destruction. To achieve this goal, we can inhibit Rpn-13, ATPase and DUBs in RP as well as the α and β subunits in CP (figure 4).

## 7. E_1_ enzymes and their inhibitors

Currently, just two E_1_ enzymes have been identified named Ubiquitin-activating Enzyme 1 (UBE1) and Ubiquitin-like modifier-activating enzyme 6 (UBA6) [34]. Only two inhibitors called PYR-41 and PYZD-4409 have recently been presented [35,36]. PYR-41 seems to inhibit the activation of the nuclear factor κ-light-chain-enhancer of activated B cells (NF-kB) by regulating the stabilization of the NF-kB inhibitors (IkB). In addition, it blocks the destruction of the tumour suppressor p53, which in turn increases the transcription of p53 [35]. PYZD-4409 induces apoptosis resulting from stress in the cancer cells and in a leukaemia mouse model and causes a delay in tumour growth [36]. Although these findings show the high potential of the E_1_ enzymes to be targeted for cancer therapy, none of these inhibitors currently have clinical efficiency because of their poor pharmacokinetic properties.

## 8. E_2_ enzymes and their inhibitors

There are 38 E_2_ enzymes in the human genome, which illustrates its specificity compared to the E_1_ enzyme [37]. CC0651 is an allosteric inhibitor for CDC34, which is a common E_2_ enzyme for cullin–ligase complexes. The fact that CC0651 causes tumour suppressors to aggregate and results in cell proliferation inhibition shows that it can become an efficient inhibitor in clinical applications [38]. However, for pharmacokinetic reasons, its development faces many problems [39]. Another potential target of cancer therapy is UBC13 (encoded by UEV1A), an E_2_ enzyme regulating the NF-kB pathway induction by forming chains depending on the ubiquitin K63. It has been shown that the NSC697923 inhibitor can inhibit the formation of K63 chains by UBC13 and also can affect the proliferation and survival of the larger B cells in lymphoma [40]. BAY-11-7082 is a well-known inhibitor of the NF-kB pathway and can inhibit IkB kinase. However, it can also inhibit UBC13 because it can prevent the binding of ubiquitin to UBC13; hence, it can inhibit the formation of the K63 chain in a similar way to the action of NSC697923 (a cell-permeable and selective inhibitor of E_2_) [41]. However, although the E_2_ enzyme inhibitors show great potential for cancer therapy, so far they have only been used in preclinical studies [33].

## 9. E_3_ enzymes and their inhibitors

**Figure 5.**
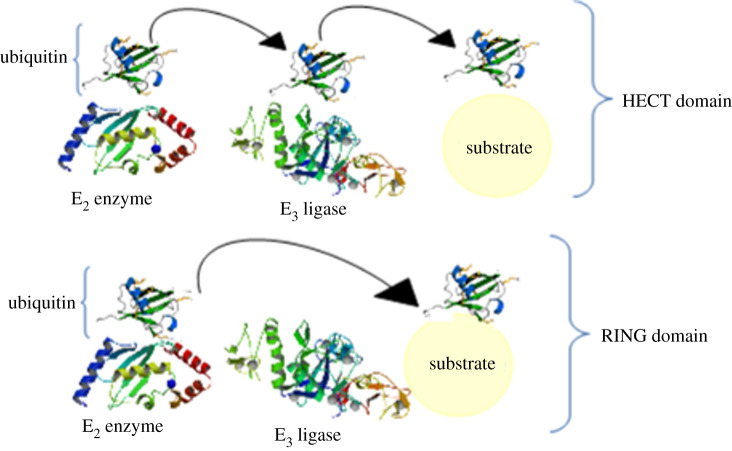
Two main classes of E_3_ ligases. HECT ligases have a C-terminal domain that accepts a ubiquitin molecule from the E_2_ enzyme by the formation of a thioester bond before the ubiquitin translocation to the substrate, and RING ligases, which have a zinc finger letting E_2_ directly transfer the ubiquitin to the substrate.

**Figure 6.**
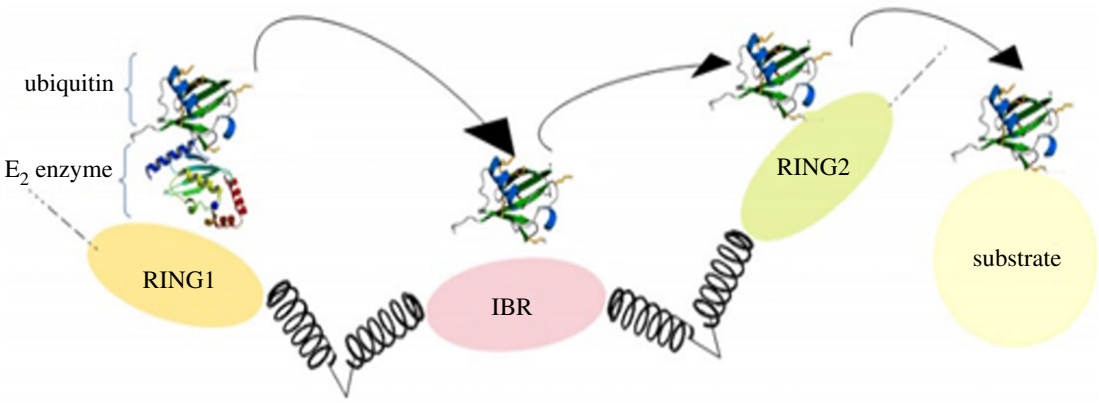
RING1-IBR (in-between-RING)-RING2 ubiquitin ligase. RBR (RING-between-RING) has two domains having elements of both RING and HECT ligases; one RING domain binds to the E_2_ while another RING domain accepts the ubiquitin molecule before its translocation to the substrate.

E3 ligases can be single peptides such as parkin, simple complexes like hetero/homodimers (e.g. mouse double minute 2 homologue (MDM2) or X-linked inhibitor of the apoptosis (XIAP) protein) and big complexes such as cullin–RING–ligase complexes or the anaphase-promoting complex/cyclosome (APC/C) [42]. Generally, there are two main classes of E_3_ ligases [43]: HECT (Homologous to the E6AP Carboxyl Terminus), in which there are 30 in the human genome, and RING ligases, which include RING and RING-like ligases and also their concomitant proteins, of which there are about 600 in the human genome (figure 5). On the one hand, HECT ligases have a C-terminal domain that accepts a ubiquitin molecule from E_2_ enzyme by forming a thioester bond before the ubiquitin translocation of the substrate [44]. On the other hand, RING ligases have a zinc finger, letting E_2_ directly transfer the ubiquitin to the substrate [45]. There is a subclass of RING ligases known as RBR (RING-between-RING), which has two domains having elements of both RING and HECT ligases; as figure 6 indicates, one RING domain binds to the E_2_, while another RING domain accepts the ubiquitin molecule before its translocation to the substrate [46]. As to the role of E_3_ enzymes in the final determination of the targeted protein, they play an important part in cell regulation. In fact, they control and regulate the key factors of apoptosis (caspases), cell growth (p21, p27 and p53), proliferation and genome stabilization (cyclin and C-Myc), immune processes (PP-L1), inflammation processes (NF-kB), and metastasis and angiogenesis (Wnt) (table 1). What is important is that the incorrect regulation of E_3_ ligases or a mutation in them results in the overexpression of oncogenes or downregulation of the tumour suppressors and, therefore, causes development of cancer. Hence, understanding the molecular goals and function of E_3_ ligases is of benefit for designing an effective method for cancer therapy [42]. As we previously mentioned, to access an efficient treatment for cancer, it is necessary to target a higher level in the UPS like E_3_ ligases [13]. Different inhibitors of these enzymes are discussed in table 1.

**Table 1.** E_3_ ligases, their notable substrates and binding partners, their function and therapeutics.

E_3_ ligase	function(s) in cell	notable substrates and binding partners	expression in cancer	cancer types	therapeutics	mechanism	model	in clinical trials	ref
E6AP	replicative immortality	p53	gain function via HPV E6	cervical, breast	CM-11 peptides	binds HECT domains	*in vitro* assay, cell culture		[47–50]
					compound 9	binds HPV E6	*in vitro* assay, cell culture		
MDM2/X	avoiding apoptosis	p53	overexpressed	many, liposarcomas	nutlins, RG7112	binds p53 binding site	*in vitro* assay, cell culture, mouse model		[51–61]
					idasanutlin (RG7388)		*in vitro* assay, cell culture, mouse model	X	
					MI-888, SAR405838	binds p53 binding site	*in vitro* assay, cell culture, mouse model	X	
					AMG-232	binds p53 binding site	*in vitro* assay, cell culture, mouse model	X	
					NVPCGM097, HDM201	binds p53 binding site	*in vitro* assay, cell culture, mouse model	X	
					JNJ-26854165 (serdemetan)	assumed to bind to RING domain of MDM2	cell culture, mouse model	X	
					ALRN-6924	stapled peptide binds MDM2 and MDMX at p53 binding site	*in vitro* assay, cell culture, mouse model	X	
SKP2	metastasis, suppressing senescence	p21, p27	overexpressed	many	compound 25	binds Skp1 binding site	*in vitro* assay, cell culture, mouse model		[62–68]
					C1, C2, C16, C20	persumed: binds Skp2, Cks1 at p27 binding site	*in vitro* assay, cell culture		
					CPDA	inhibits Skp2–Skp1 binding	*in vitro* assay, cell culture		
					NSC689857, NSC681152	inhibits Skp2–Cks1 binding	*in vitro* assay		
Fbxw7	genome instability, angiogenesis	Cyclin E, mTOR	downregulated or dominant-negative mutant	many; endometrial, cervical, blood, leukaemia	oridonin	stabilizes Fbxw7, increases the activity of kinase Gsk-3, modulates proteasomal degradation of BCR-ABL in leukaemia, inhibits Nrf2 pathway	*in vitro* assay, cell culture		[69–71]
β-TrCP	suppressing senescence, chronic inflammation	IkB, β-catenin, Wee1, Cdc25 a/b	overexpressed (in some tissue)	many	eriflorin	inhibits β-TrCP1 binding to the substrate	*in vitro* assay, cell culture		[62,72]
					GS143	persumed: inhibits binding of β-TrCP1 and p-IkBa	*in vitro* assay, cell culture		
Cdc20	genome instability	Cyclin A/B, securin	overexpressed	pancreatic, lung, gastric	appin	binds to D-box binding site of Cdc20	*in vitro* assay, cell culture		[63,67,73]
					ProTAME	inhibits formation of APC/C–Cdc20, Cdh1	*in vitro* assay, cell culture, mouse model		
Cdh1	genome instability	Cdc20, PIK1, Aurora kinase A/B	under-expressed	many	ProTAME	inhibits formation of APC/C–Cdc20, Cdh1	*in vitro* assay, cell culture, mouse model		[74,75]
XIAP	metastasis, avoiding apoptosis	p62, Caspases 3,9,7	overexpressed	many	LCL161	binds to BIR3 domain of XIAP	*in vitro* assay, cell culture, mouse model	X	[76–79]
					AEG35156	XIAP antisense oligonucleotide	cell culture	X	
Park2	metastasis, altered metabolism, immune system evasion	cyclin D/E, Cdc20/Cdh1, tubulin	under-expressed	breast, pancreatic, colorectal, ovarian					[80]
SPOP	genome instability, immune system evasion	PD-L1, androgen and estrogen reseptor	downregulated or dominant-negative mutant	prostate, endometrial, kidney	palbociclib	Cdk4 phosphorylates SPOP, destabilizes PD-L1	*in vitro* assay, cell culture, mouse model		[81,82]
					compound 6b	binds to substrate pocket	*in vitro* assay, cell culture, mouse model		

### 9.1. Tumour protein p53

Tumour protein p53 (Tp53) is a transcription factor known as a regulator of cell functions and proliferation and a controller of the cell cycle, responding to DNA damage and apoptosis [42]. In addition, p53 has an anti-cancer property through inhibiting angiogenesis. When tumours grow, they need angiogenesis to supply nutrients. Therefore, p53 decreases tumour growth through involvement in the creation of hypoxia-inducible factors HIF1, HIF2, which impact angiogenesis, inhibiting the production of angiogenesis inducing factors and increasing the production of angiogenesis inhibitors like arresten [83,84]. Mechanical signals cause HIF-1α and HIF-2α to affect the amounts of p53 in stem cells so that HIF-1α stabilizes p53, whereas HIF-2α suppresses it [85]. The α subunit of HIF becomes hydroxylated in its proline residue by HIF proline hydroxylase, which results in its ubiquitination by an E_3_ ligase named VHL (von Hippel-Lindau tumour suppressor) and its rapid degradation by the proteasomes [86]. What increases the HIF factor in the hypoxic condition is the inhibition of prolyl hydroxylase, which affects the amounts of p53 [87].

In unstressed cells, the amount of p53 is kept low by its continuous destruction. To this end, one protein named MDM2, which is called HDM2 in humans, binds to p53, prevents its action, and transfers it from the nucleus to cytosol. MDM2 also acts as a ubiquitin ligase and conjugates the ubiquitin covalently to p53 and, therefore, marks p53 to be destroyed by proteasomes. However, p53 ubiquitination is reversible. Interestingly, p53 becomes activated by MDM2 activation, which sets up a feedback loop. The amount of p53 can show oscillations in response to certain stresses, thus indicating whether the cells survive or die [88]. When activation of p53 is necessary, MI-63 (a small molecule inhibitor of MDM2–p53 interaction) binds to MDM2 and reactivates p53 [89]. Various combinations stabilize p53, for example, by deubiquitinating it, which is necessary for p53 activity against cell stress [90]. A special ubiquitin protease called USP7 (or herpes virus-associated ubiquitin-specific protease (HAUSP)) can remove the ubiquitin from p53 and protect it from destruction by the proteasome. Recent studies have shown that HAUSP is usually located in the nucleus, although some of it may be found in the cytoplasm and mitochondria. Besides, its overexpression causes the stabilization of p53. However, not only does depletion of HAUSP fail to decrease p53 levels, but it actually increases its levels since HAUSP binds to MDM2 and deubiquitinates it. In unstressed cells, HAUSP seems to bind with MDM2 more easily than p53.

Another factor that stabilizes p53 is ubiquitin carboxyl-terminal hydrolase 10 (USP10) (a deubiquitinase). In unstressed cells, USP10 is located in the cytoplasm, it deubiquitinates cytoplasmic p53 and reverses the ubiquitination of MDM2. Following DNA damage, it becomes located in the cytoplasm and increases the stabilization of p53. Notably, USP10 does not bind to MDM2 [91]. Another way to stabilize p53 is by phosphorylating its N-terminal domain, preventing the binding of MDM2 to it. Some proteins such as pin1 bind to p53 and cause a change in its structure, which results in more protection against MDM2. Phosphorylation of p53 also leads transcription factors such as P300 and P300/CBP-associated factor (PCAF) to be bound to p53 and then acetylate its C-terminal domains. However, deacetylase enzymes such as sirt1 and sirt7 can deacetylate p53 and, therefore, block apoptosis [92]. Some oncogenes can also inhibit the activation of MDM2 by increasing the transcription of those proteins bound to it.

Additionally, another E3 ligase that affects p53 is called E_3_ ubiquitin ligase E6-associated protein (E6AP) or Ubiquitin-protein ligase E3A (UBE3A). It is a HECT ligase with a molecular weight of about 100 kDa, which was discovered as an interaction between human papillomavirus E6 protein and p53. Neither E6AP nor E6 has a strong relation with p53, but as a complex, E6/E6AP can bind to p53 and change the properties of the E6AP substrate, which lets E6AP ubiquitinate p53 and target it for degradation [93]. Significantly, E6AP may have a role in cervical cancer caused by the human papillomavirus [94].

### 9.2. SCF complex (Skp, Cullin, F-box containing complex)

#### 9.2.1. SKP2

The SCF complex is a multiunit playing several roles in cell regulation. Cullin1 (Cul1) is the main substrate of this complex, which ubiquitinates targeted proteins in their N-terminal domains. Notably, it uses two adapter proteins to bind to the substrate; Cul1 directly binds to S-phase kinase-associated protein 1 (SKP1), then binds to 70 F-box proteins, which directly binds to the substrate [95]. F-box proteins are those having at least one F-box domain. The F-box domain is a structural protein having 50 amino acids, which intermediates the interaction between the proteins. The F-box protein in SKP2 has three α helices and directly binds to the SKP1 protein of SCF [96]. Cul1, in its C-terminal domain, binds to the protein adapters named RING-box protein 1 (Rbx1) and RING-box protein 2 (Rbx2), which will bind to an active E2 enzyme [97,98].

The S-phase kinase-associated protein 2 (SKP2) is an F-box protein that is more active in the S-phase [99]. In the S-phase, E cyclin binds to the ubiquitinated and phosphorylated p27 using the Cyclin-dependent kinase 2 (Cdk2) complex [100]. p27 is a Cdk inhibitor that in humans is coded by a gene called CDKN18 [101]. It also binds to the Cdk2–E cyclin and Cdk4–D cyclin complex and inhibits their action, hence controlling the cell cycle at the G1 point. p27 degradation reverses Cdk2–E/A cyclin complex inhibition, thus letting the cell enter S-phase and become prepared to enter mitosis [102].

However, another target of SKP2 is p21. p21 is known as a Cdk1 inhibitor, which is able to inhibit all Cdk/cyclin complexes [103], although it firstly inhibits Cdk2 [104,105]. Additionally, it is the main target of p53 and, therefore, collaborates with DNA damage for cell cycle arrest [106–108]. In some cases, to reinforce the SKP2 binding to the substrate, it is necessary to use a helper protein called CKS1 [109]. SKP2 can reinforce the transcription of C-Myc as well as its degradation [110]. It is shown that C-Myc represses the p21 promoter [111]. Interestingly, acetylation of p300 by SKP2 changes the location of SKP2 from the nucleus to the cytoplasm, resulting in an increase in cell proliferation and tumour regeneration [112]. SKP2 mutations have recently been reported in several cancers, including blood, colorectal, bone, ovarian and cervical [109,113].

#### 9.2.2. Fbxw7

F-Box and WD Repeat Domain Containing 7 (Fbxw7) (in yeast, Cdc4) have a homodimeric domain and an F-box domain that binds to SKP1. The structure binding to the substrate is a β-propeller structure formed from eight WD40 repeats located on Fbxw7 [114]. Binding of the substrate to Fbxw7 depends on the WD40 arginine contact with phosphorylated region of the substrate [114]. What is important is that the mutations preventing substrate binding, especially in the arginine of the WD40 region, are usually seen in tumour samples [115]. Due to the homodimerization of Fbxw7, these mutations may have a dominant-negative effect [116]. This effect relates to the fact that the mutant Fbxw7 can effectively bind to the substrate while it is unable to ubiquitinate it [117]. Notably, mutation of Fbxw7 is common in several cancers such as blood and bile duct [118].

One of the best substrates for Fbxw7 is E cyclin [119], whose degradation and ubiquitination depend on being phosphorylated by Cdk2 and glycogen synthase kinase 3 (GSK3) [120]. Interestingly, dimerization of Fbxw7 can change its ability to bind to E cyclin and other substrates [121]. Other targets of Fbxw7 include transcriptor factors such as Notch1, C-JUN and Myc-C [122], proteins bound to DNA [123] and mTOR (the mammalian target of rapamycin) protein. It is noteworthy that the large T antigen of SV40 (the simian vacuolating virus 40 or simian virus 40), which has a CDP domain, can inhibit Fbw7-driven cyclin E turnover [43,124].

#### 9.2.3. β-Transducin repeat-containing protein

β-Transducin repeat-containing protein (β-TrCP) play an important role in regulating the controlling points of the cell cycle. It also inhibits the action of Cdk1 by destroying CDC25A (M-phase inducer phosphatase 1) in cooperation with the Chk1 in response to genotoxic stress [125,126] and results in blocking of cell cycle development before DNA repair is completed. During DNA repair, β-TrCP targets claspin with a phosphatidylinositol 4-kinase (PIK1)-dependent method [127–129]. In addition, β-TrCP is known as an important factor in protein translation, cell growth and cell survival. Indeed, in response to mitogens, programmed cell death protein 4 (PDCD4), which is an inhibitor of the translation initiation factor eIF4A (eukaryotic initiation factor-4A) is destroyed in a β-TrCP and S6 kinase beta-1 (S6K1)-dependent condition. This leads to cell growth and elongation of protein translation [130]. Another target of β-TrCP playing a role in protein translation is eukaryotic elongation factor-2 kinase (eEF2K), which inhibits the elongation of protein translation by phosphorylating eukaryotic elongation factor 2 (eEF2) and also decreasing its attraction to the ribosome [131]. In addition, β-TrCP helps mTOR and Casein kinase 1α (CK-1α) to destroy DEPTOR (an inhibitor of mTOR) and produces an automatic reinforcement loop for the complete promotion of mTOR activation [132–134]. At the same time, β-TrCP destroys an apoptosis protein named BimEL (the major splice variant of Bcl-2-interacting mediator of cell death) to promote cell survival [135]. Moreover, in the same tissue, β-TrCP acts as an oncoprotein. High levels of β-TrCP expression are found in cancers, including colorectal [136], pancreatic [137], hepatoblastoma [138] and breast cancers [139].

### 9.3. APC/C complex

Enhanced cell cycle performance and successful mitosis rely on the coordination between cyclin function and degradation [140]. The disruption of this coordination can result in mitosis mistakes, unconsciousness and cancers [141]. While Cdk 1/2 mediates cell entrance to mitosis, the continuation of mitosis and the cell's exit from mitosis depend on APC/C [42]. Cell division cycle 20 (Cdc20) is a basic regulator of cell division, which in humans is coded by the CDC20 gene [142]. According to current knowledge, its most important function is activation of APC/C. The protein complex APC/C–Cdc20 has two basic downstream goals. First, it targets the securin for destruction, which in turn leads to cohesion degradation and, therefore, results in chromatid separation. It also targets the S and M phase cyclins for destruction, letting the cell exit from mitosis.

Notably, another protein that plays a complementary role in the cell cycle is named Cdh1, which is a homologue with Cdc20. As a regulatory protein, Cdc20 cooperates with lots of other proteins at different cell cycle points. In summary, this protein is necessary for two microtubule-dependent processes: nuclear motivation before anaphase and chromosome separation [143].

### 9.4. Other E_3_ ligases

#### 9.4.1. XIAP

XIAP is an E_3_ ligase from the IAP family known to have three IAP baco virus N-terminal domains and a RING C-terminal domain [144]. IAPs, such as XIAP, play the main role in regulating cell response to apoptosis. XIAP is expressed in several cancers, especially kidney and skin cancers [145,146]. The XIAP binding region between BAK1-interacting receptor-like kinase 1 (BIR1) and BAK1-interacting receptor-like kinase 2 (BIR2) includes the active site and inhibits caspases 3 and 7 [147]. Furthermore, the BAK1-interacting receptor-like kinase 3 (BIR3) domain of XIAP binds to caspase 9 and inhibits its activation [148]. Moreover, XIAP ubiquitinates caspase 3, caspase 9 and caspase 7 target them for destruction by the proteasome [145]. Ultimately, in addition to its role as an E_3_ ligase, XIAP also acts as a neddylator, by which it inhibits the activation of caspases [149].

Neddylation is a process in which a ubiquitin-like protein, NEDD8, becomes conjugated with the substrate. This process is similar to ubiquitination, although it is dependent on its own E_1_ and E_2_ ligases. NEDD8 binds to the substrate by forming the isopeptide bond between the glycine of the NEED8 C-terminal domain and the substrate's lysine. Neddylation of the substrate results in structural change that may prevent molecular motivation and block conjugation of different substrates to it. In addition, it can cause the proteins, which are usually bound to the substrate, to become incompatible with it. For example, Cullin-Associated NEDD8-Dissociated Protein 1 (CAND1) could not bind to a neddylated protein [150].

#### 9.4.2. Park2

Park2 (PARKIN) is an RBR-E_3_ ligase, and hence, as mentioned before, it has both HECT and RING properties [151]. In cancer, the location of park2 is usually eliminated [152]. In the mouse model, elimination of park2 results in liver cancer [153]. Additionally, it can cause colorectal cancer [154]. Moreover, park2 plays the main role in mitophagy [155] that may impact cell redox [156], proliferation and metastasis [157]. The role of park2 in regulating the cyclin level is an important factor. Park2 destroys the D [158] and E [159] cyclins by a Cul1-dependent method [160]. The mutation of park2 that takes place in cancers results in the stabilization of these cyclins in the G1/S phase, which causes an increase in the number of the cells being in the S and G2/M phases [159,160]. Also, it can increase the speed of cell proliferation [158]. Moreover, during mitosis, park2 cooperates with Cdc20 and Cdh1 by an APC/C-dependent method [161]. Furthermore, park2 ubiquitinates HIF-1α and, therefore, helps in cell migration. Consequently, its elimination causes tumour metastasis in mouse models [162].

#### 9.4.3. Speckle Type BTB/POZ protein

The Speckle Type BTB/POZ protein (SPOP) is a Cul1 adapter that is mutated in 10% of prostate cancers [163]. Moreover, SPOP has three main regions: a MATH N-terminal for recognizing the substrate [164], a BTB domain for dimerization and interaction with cul 3 and a BACK domain for assembling SPOP dimers as oligomers [165]. Interestingly, oligomerization of SPOPs increases their binding to the substrate and increases the substrate ubiquitinated by SPOPs [166]. Owing to the SPOP role in the regulation of the proteins involved in cell protection, a mutation in the MATH domain blocks the binding of SPOP to the substrate, hence it causes cancer development [167]. In addition, it plays a role in immunotherapy by ubiquitinating and degrading programmed death-ligand 1 (PD-L1) [168]. What is important is that mutant SPOPs cannot ubiquitinate PD-L1 that lets the tumour cells grow [167]. Similarly samples from pancreas cancer with mutant SPOP had high levels of PD-L1, showing the role of SPOP in immune system invasion [169]. Some of the other proteins targeted by SPOP are: proto-oncogene DEK [170], deSUMOlyase SENP7 [171], C-Myc [172], Cdc20 [173], phosphatases PTEN and Dusp 7 [165], Gil 2 and Gil 3 [174,175] and transcription factors BET (BRD 2–4) [176–178].

## 10. Proteasome inhibitors

### 10.1. 20S core (CP) inhibitors

In multiple cancers consisting of hepatocellular carcinoma (HCC), breast cancer, colorectal cancer and lung cancer, the proteasome subunits showed maladaptive expression [179]. For instance, the proteasome beta subunit 4 (PSMB4), a subunit of the CP complex, is upregulated in epithelial ovarian cancer in which clinicopathological characteristics and worse prognosis occur owing to the overexpression of PSMB4 [180]. Furthermore, the 26S proteasome non-ATPase regulatory subunit 10 (PSMD10), another well-studied oncoprotein as a valuable biomarker for recurrence and survival, is often overexpressed in HCC and enhances HCC invasiveness and metastasis [181]. Moreover, it regulates the balance between apoptosis and cell cycle by degrading retinoblastoma protein transcriptional corepressor 1 (RB1) and Tp53 [182,183].

#### 10.1.1. α subunit inhibitors

Although it seems that proteasome alpha subunits (PSMAs) take part in the malignant progression of human cancers, in most of them, the expression patterns and prognostic values of individual PSMAs remain elusive. Even though seven alpha subunits (PSMA1–7) have been known, only several of them have proved to have an association with cancers. For instance, pulmonary neuroendocrine tumours have increased PSMA1 and PSMA5 mRNA expression compared to normal tissues. Moreover, susceptibility to lung cancer has an undeniable relationship with PSMA4 polymorphisms, so an increased level of PSMA4 has a significant role in the regulation of cell proliferation and apoptosis in lung cancer. PSMA7 can also take part in the development of HCC by destroying several proteins playing a role in replicating the hepatitis B virus. Furthermore, colorectal cancer has shown overexpressed levels of PSMA7 that are meaningfully related to the cancer patient's prognosis. Interestingly, it has been shown that PSMA7 depletion in colorectal cancer cells leads to decreased cell invasion and migration [179].

**Figure 7.**
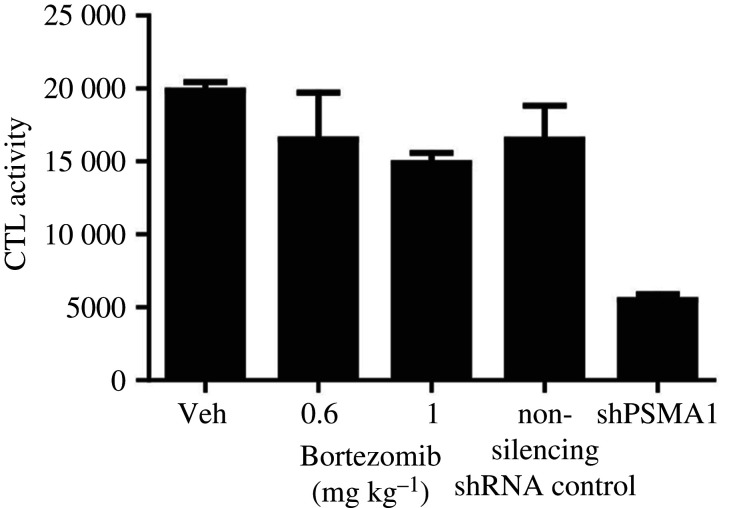
Comparison between the inhibition activity of Bortezomib and shPSMA1 on chymotrypsin proteasome. PSMA1 knockdown showed more inhibition compared to Bortezomib. CTL, chymotrypsin-like; Veh, vehicle. Adapted from [184]. Copyright © 2013 Public Library of Science.

Accordingly, PSMAs are involved in numerous human cancers; however, inhibitors of these subunits are not used frequently. The only report of their utilization to date was by Cron *et al*. in 2013 [184], who investigated whole-genome RNAi screens to recognize knockdowns that increase non-small cell lung cancer (NSCLC) cytotoxicity most reproducibly, the treatment of which fails in a majority of locally advanced patients despite optimal radiation therapy (RT), chemotherapy and/or surgery. PSMA1 is one of several proteasome subunits recognized by these screens among top hits, and was the topmost one. Some of the synergistic impacts of radiation and proteasome inhibition are as follows: a 50% reduction in the expression of NF-kB-inducible homologous recombination (HR) genes BRCA1 (breast cancer type 1 susceptibility) and FANCD2 (FA complementation group D2), an 80–90% reduction in HR and a decrease in FANCD2, BRCA1 and RAD51 ionizing radiation, which induced foci. Notably, to knockdown PSMA1, they use doxycycline. Interestingly, Cron *et al*. observed more proteasome inhibition after PSMA1 knockdown in comparison with the utilization of Bortezomib (figure 7), which may be due to poor tumour drug penetration of Bortezomib [184]. Despite this advantage, more investigation is needed to use these inhibitors clinically.

#### 10.1.2. β subunit inhibitors

Generally, there are five main classes of these kinds of proteasome inhibitors: peptide aldehydes, peptide boronates, epoxomicin and epoxyketones, lactacystin, β-lactone and vinyl sulfones. Most of these inhibitors have an inhibition effect on the β_5_ subunit of proteasomes because β_1_ and β_2_ inhibitions are not as effective as they should be. However, inhibition of β_5_ is much more effective.

Peptide aldehydes (e.g. MG-132) were the first proteasome inhibitors to be discovered [185]. However, these inhibitors have several deficiencies; for example, in cell culture, MG-132 becomes rapidly oxidized and, therefore, converted to an inactive acid [186]. In comparison to peptide aldehydes, peptide boronates are more effective inhibitors. The dissociation rate of peptide boronates is slower and the reaction is irreversible [186]. What is more, they do not become oxidized; hence, they are much more stable [186]. Another class of proteasome inhibitors is Epoxyketone, and the most well-known Epoxyketone inhibitor is Carfilzomib [187]. Lactacystin is a nanopeptide inhibitor [187]. What is interesting is that it does not directly inhibit the proteasome. At neutral pH, it converts to clasto-lactacystin-β-lactone, reactivating the proteasome [187]. Ultimately, vinyl sulfones are another class of proteasome inhibitors whose synthesis is easy and inexpensive [187].

Three proteasome inhibitors named Bortezomib, Carfilzomib and Ixazomib are currently confirmed by the FDA (US Food and Drug Administration). At first, Bortezomib was approved in 2003 for those patients having relapsed MM [188]. Nowadays, its usage has been expanded for new MM patients and for the treatment of mantle cell lymphoma (a rare form of non-Hodgkin lymphoma having a usual scatter pattern of small lymphocytes and small slit cells) [189]. Generally, three models are well known; inhibiting NF-kB by stabilization of IkB, activating the response of misfolded proteins by inhibition of proteasome due to high endoplasmic reticulum (ER) stress, and finally, stabilization of the apoptosis proteins such as BAX (BCL2 associated X, apoptosis regulator) and NOXA (BH3-only member of the BCL-2 family) [39,188,190,191].

Carfilzomib was approved in 2012 by the FDA for those patients having relapsed MM and previously cured by Bortezomib [189,190]. This inhibitor binds reversibly to the proteasome and inhibits its activation up to 80%. As mentioned previously, it is used when Bortezomib is not effective [39]. In 2015, another proteasome inhibitor was approved by the FDA named Ixazomib. It was used in combination with lenalidomide and dexamethasone. In contrast with Bortezomib and Carfilzomib, Ixazomib is available orally [192]. There are two more proteasome inhibitors that are in clinical trials: Morizomib and Oprozomib. Morizomib is a kind of β-lactone inhibitor. In high concentrations, it can also inhibit the β_1_ subunit [193]. What is important is that Morizomib can overcome the resistance to Bortezomib and Carfilzomib [194]. Oprozomib is another proteasome inhibitor that is in the class of epoxyketone inhibitors [187]. All of these inhibitors inhibit the β_5_ subunit of the proteasome (chymotrypsin).

**Figure 8.**
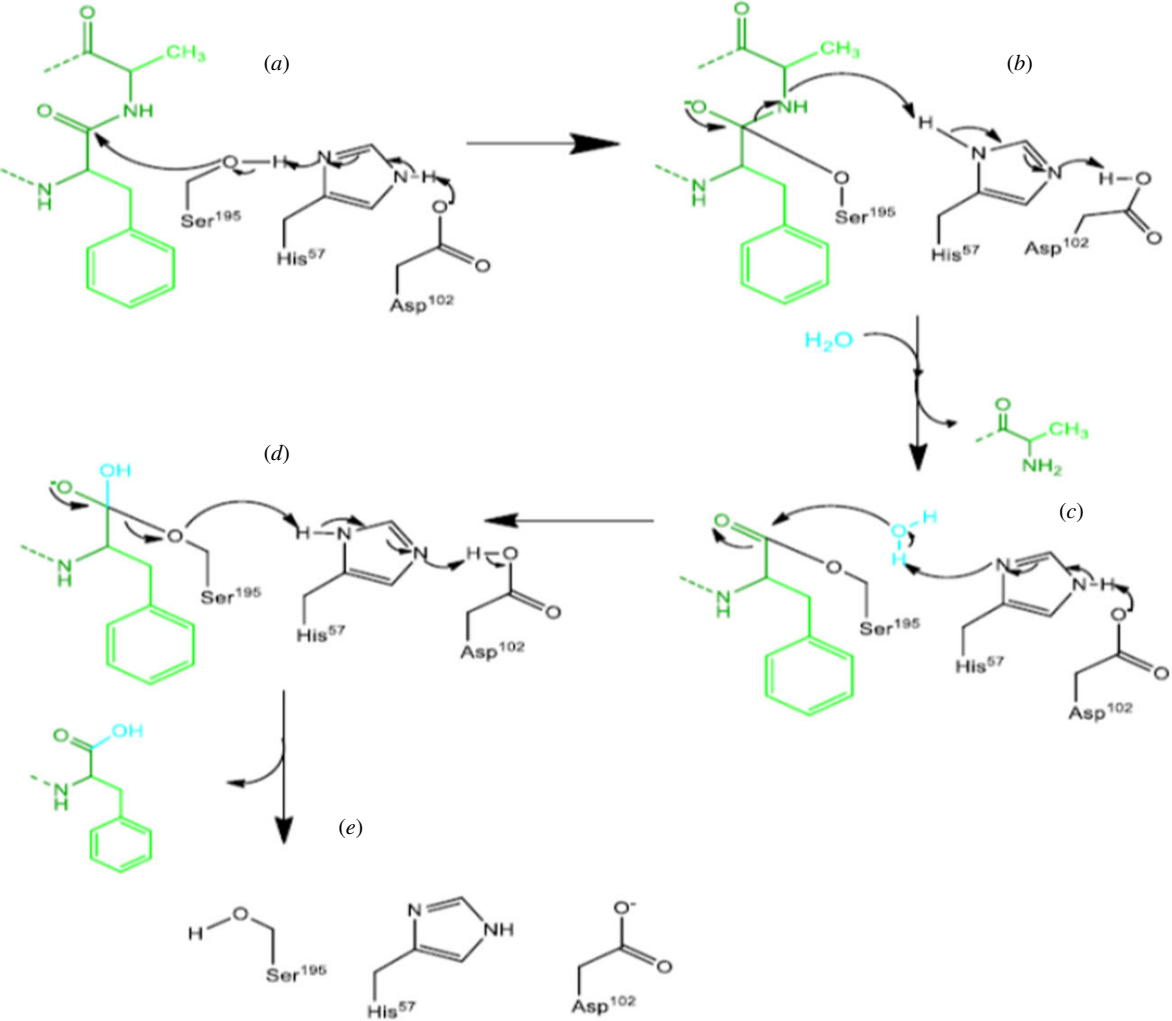
The process of protein proteolysis by chymotrypsin. (*a*) The substrate becomes acylated to form an acyl-enzyme intermediate. The hydrogen of the aspartate binds to the N-δ hydrogen of histidine. (*b*) The sidelong chain of the serine acts as a nucleophile and binds to the carbonyl carbon of the substrate's main chain. (*c*) Ionization of carbonyl oxygen becomes stabilized by the formation of two sidelong hydrogen bonds to the N-hydrogens of the main chain. These reactions result in a tetrahedral combination and cause the peptide bond to be broken down. (*d*) Formation of an acyl-enzyme intermediate bonded to serine and causes the new N-terminal of the protein to be broken down and separated. Moreover, in the second step of the reaction, one water molecule becomes activated by histidine and acts as a nucleophile. The oxygen of the water attacks the carbonyl carbon of the acyl group of serine, which results in the formation of a secondary tetrahedral combination, regeneration of the OH group of serine and the release of a proton as well as a protein with a newly formed C-terminal domain.

The reaction between chymotrypsin and substrate takes place in two steps: a primary step at the start of the reaction and a steady-state phase following Michaelis–Menten kinetics. The first step is hydrolysis, which in turn is done in two steps: (i) acylation of the substrate to form an acyl-enzyme intermediate and (ii) deacylation of the enzyme to its first form. These events occur by the coordinate action of three amino acids in a catalytic triple [195]. The aspartate's hydrogen bonds to the N-δ hydrogen of histidine, causing an increase in the p*K*_a_ of its fourth nitrogen. Hence, serine becomes deprotonated, which means serine loses its proton. This deprivation causes the serine's sidelong chain to act as a nucleophile and bind to the carbonyl carbon of the main chain of the substrate, which had electron deficiency. The ionization of carbonyl oxygen becomes stabilized by the formation of two sidelong hydrogen bonds to the main chain's N-hydrogens. These reactions result in a tetrahedral combination and lead the peptide bond to be broken down. The formation of an acyl-enzyme intermediate bonded to serine causes the new N-terminal of the protein to be broken down and separated. Moreover, in the second step of the reaction, one water molecule becomes activated by histidine and acts as a nucleophile. The oxygen of the water attacks the carbonyl carbon of the acyl group of serine, which results in the formation of a secondary tetrahedral combination, regeneration of the OH group of serine and the release of a proton, as well as a protein with a newly formed C-terminal domain (figure 8) [195]. In these reactions, if the aspartate amino acid of the chymotrypsin binds to an inhibitor, the rest of the hydrolysis route will not continue; therefore, the proteasome becomes inactivated. For example, the bromine atom in the Bortezomib can bind to the oxygen of the aspartate and inactivate it.

**Figure 9.**
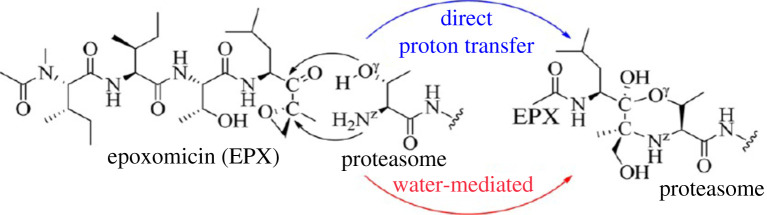
The suggested mechanism for the reaction of epoxomicin (EPX) with proteasomes. Possible mechanisms for this reaction are via five steps. (i) Proton transfer process, activating Thr1-Oγ directly via Thr1-Nz to form a zwitterionic intermediate. (ii) Nucleophilic attack on the carbonyl carbon of EPX by the negatively charged Thr1-Oγ atom. (iii) Proton transfer from Thr1-Nz to the carbonyl oxygen of EPX. (iv) Thr1-Nz attacks the carbon of the epoxide group of EPX, along with the epoxide ring-opening (SN2 nucleophilic substitution) so that a zwitterionic morpholino ring is formed between residue Thr1 and EPX. (v) The product of the morpholino ring is generated by another proton transfer. Adapted from [196]. Copyright © 2012 American Chemical Society.

It is noteworthy that the above findings have also been confirmed by computational methods so that Wei *et al*. used first-principles quantum mechanical/molecular mechanical (QM/MM)-free energy (QM/MM-FE) calculations to illustrate the first detailed systematic computational study on the reaction mechanism of the proteasome with Epoxomicin inhibitors (EPX) [196]. In this study, they carried out MD simulations and QM/MM-FE calculations to further explore the subsequent chemical reaction mechanism of EPX with proteasome in the β5 catalytic site (chymotrypsin). According to figure 9, these computational studies demonstrate that the possible mechanisms for this reaction are via five steps presented in [196].

However, several inhibitors exist acting on other mechanisms. For instance, bioinformatics studies of Di Dato *et al*. [194] demonstrated that the cationic porphyrins as new lead structures could be used for the development of a novel class of multifunctional inhibitors. Indeed, docking methods and NMR measurements show that cationic porphyrins act as a reversible inhibitor of the human 20S proteasome by using the interactions of the positively charged *N*-methyl-pyridyl moieties with the negative residues on the α-ring in proximity to the gate channel [197]. Notably, these highly versatile inhibitors conjugate to the parent frame with variously charged moieties, which in turn devote special properties to the molecule. It confirms they may be fine-tuned and that this can be correlated to the key role of electrostatics in driving porphyrin/proteasome interactions.

### 10.2. 19S regulatory cap inhibitors

#### 10.2.1. Ubiquitin receptor inhibitors

Rpn-10 and Rpn-13 are two ubiquitin receptors of the 26S proteasome in the 19S RP. Rpn-10 (originally called S5) was first recognized in humans as a proteasome subunit in 1994 [198] and Rpn-13 was first reported in 2006 [199–201]. As mentioned previously, these ubiquitin receptors are exactly located at the apex of the 26S proteasome, making it possible for polyubiquitinated substrates to be accessibly captured and to be degraded. While Rpn-13 contains one ubiquitin-interacting surface, Rpn-10 has two ubiquitin-interacting motifs (UIMs), which can bind to ubiquitin chains at the same time but at different affinities [202]. Since Rpn-13 is unnecessary in healthy cells and is overexpressed in a variety of cancers, including ovarian, MM and gastric cancer, it has been more noteworthy than Rpn-10 up to now (electronic supplementary material, S2) [203–205]. Until now, only two inhibitors of Rpn-13 have been known: a covalent irrevocable chalcone named RA190 and a non-covalent revocable peptoid named KDT-11.

Notably, the optimization of activity and solubility of several chalcones such as AM114 that is known to be inhibited by ubiquitin-mediated protein degradation led to the discovery of RA190 in 2013 [206–208]. RA190 seems to trigger the unfolded protein response (UPR) and cause accumulation of polyubiquitinated proteins, resulting in ultimate cell apoptosis. Bortezomib-resistant cell lines are an important problem that can be treated by this mechanism of action. Additionally, it has promising anti-cancer activity towards cervical, MM and ovarian cancers [206,208]. In 2015, a one-bead one-compound (OBOC) peptoid library screen of 100 000 compounds resulted in the discovery of the second well-known inhibitor of Rpn-13, called KDT-11 [209]. KDT-11 acts like RA190 since it is a well-known 20S CP β5 inhibitor, which has also appeared to cause the accumulation of polyubiquitinated proteins in MM cells that leads ultimately to cell apoptosis. A competitive fluorescence polarization (FP) assay has suggested that KDT-11 contrasts with RA-190 in regard to their surface binding. Moreover, KDT-11 could rather destroy the Uch3–Rpn-13 interaction or bind to a novel surface on Rpn-13. In conclusion, despite the advantage of KDT-11 over RA190, which is the selectivity of KDT-11 for Rpn-13 in MM cells, improvement of its physical properties needs more *in vivo* investigation [210].

#### 10.2.2. ATPases inhibitors

Rpt1–6 are six distinct ATPases in the human proteasome that form a heterohexameric ring-like structure [211] located on the top of the 20S CP's alpha subunits (figure 2) [212]. As mentioned before, this ATPase ring can unfold and shuttle the proteasome's selected proteins for degradation via 20S CP. In 2007, screening 30 000 compounds led to the identification of a peptide called RIP-1 [213]. This compound was shown to have inhibitory effects on Rpt1–6, especially Rpt4. However, more investigations are required to reveal that targeting Rpt4 is a viable mechanism of toxicity.

Besides the Rpt subunits, p97 (VCP, for valosin-containing protein) is another ATPase that is greatly involved in the UPS [214]. The homohexameric p97 protein complex differs from the Rpt subunits in that the former appears to ‘pick’ ubiquitinated proteins from inside the cell and transport them to the 19S RP, whereas the latter does not [215,216]. In addition, its upregulation in multiple diseases and cancers highlights it as a potential therapeutic target [217,218]. Four inhibitors are known to inhibit p97. DBeQ is a selective and reversible p97 ATPase inhibitor that can block endoplasmic reticulum-associated degradation, modulate the autophagy pathway and enhance the activation of cascades 3 and 7 in cancer cells. ML240 is an ATP-competitive inhibitor of p97 ATPase, which is similar to DBeQ, can promote activation of caspases 3 and 7 (in multiple colon cancer cells) and inhibit the endoplasmic reticulum-associated degradation (ERAD) pathway. It can also induce the accumulation of LC3-II (a standard marker for autophagosomes) and modulates autophagosome maturation. Another inhibitor is NMS873, which is a potent and selective p97 ATPase allosteric inhibitor and is able to activate the unfolded protein response and impair autophagosome maturation. It can also present antiproliferative activity in cancer cells [219]. Ultimately, another inhibitor that has recently been suggested is xanthohumol, which is a polyphenol [220].

### 10.3. Deubiquitinating enzymes (deubiquitinases) inhibitors

Deubiquitinating enzymes (DUBs) (also deubiquitinases) remove ubiquitin from target proteins and, therefore, reverse the effect of E3 ligases. Moreover, they can also take part in ubiquitin recycling, maturation and editing [221–223]. Remarkably, about 100 DUBs exist that can be encoded by the human genome. Cysteine proteases and zinc metalloproteases are two main classes of DUBs based on the mechanism of enzymatic cleavage. However, in another division based on sequence and domain conservation, DUBs group into six subfamilies: Machado–Joseph disease protein domain proteases (MJDs), monocyte chemotactic protein-induced protein (MCPIP), ubiquitin carboxy-terminal hydrolases (UCHs), ubiquitin-specific proteases (USPs), ovarian-tumour proteases (OTUs) and JAB1/MPN/Mov34 metalloenzyme (JAMM)/Mpr1, Pad1 N-terminal (MPN) domain-associated metallopeptidases (JAMMs) [222–225].

Conspicuously, the most numerous DUBs are USPs, which have approximately 60 proteases in humans and have sizes ranging from 50 to 300 kDa [223]. USPs consist of a huge subdivision of proteins that have relevant DUB activity. In particular, owing to extensive gene mutations and USPs' aberrant expression in different types of cancers, USPs are considered potential anti-cancer targets. Also, interest in developing USP-specific inhibitors as anti-cancer therapeutic agents is increasing [226,227].

Up to now, several inhibitors have been developed that target different DUBs. For example, 8-mercapto-*N*-((tetrahydro-3-furanyl)methyl)-4-quinoline carboxamide, LND-57444, VLX1570, ML323, (ADC-01,ADC-03, HBX41108,HBX19818,P5091,P22077), 9-(ethoxyimino)-9H-indeno(1,2-b)pyrazine-2,3-dicarbonitrile, WP1130, Mitoxantrone and GSK2643943A are able to inhibit PSMD14, UCHL1, UCHL5 and USP14, USP1, USP7, USP8, USP9X, USP11 and USP20, respectively. Markedly, all of these inhibitors are in the preclinical stage except for VLX1570, which is in the clinical trial phase [222].

However, among all of these DUBs, the most targeted part of the RP is Rpn11. Remarkably, it is a metalloprotease that is directly located above the translocation pore and removes ubiquitin chains from the substrates targeted for degradation [228]. Particularly, every mutation that disrupts its catalytic activity can block the substrate degradation and ultimately cause cell death [229]. Capzimin is the first-in-class selective inhibitor of Rpn11 recently developed by Li *et al*. in 2017 [230]. In principle, what capzimin treatment does is stabilize targeted substrates and inhibit proliferation in several tumour cell lines [230]. This anti-tumour activity introduces Rpn11 inhibition as a productive alternative for treating malignancies.

## 11. Future perspectives

Although the proteasome–ubiquitin system has the potential to find an efficient treatment for cancer, until 2019, it was used mostly for treating MM. However, in 2019, Rashid *et al*. reported that this system's inhibitors can be efficaciously used for the treatment of brain cancer. This report opened a new vision in this field [231]. Therefore, different ways for inhibiting this system in addition to ways to improve these inhibitors have been suggested up to now.

Interestingly, several chemical changes using computational methods can introduce new inhibitors with much more efficiency. Multiple ways exist to improve the properties of inhibitors. For example, the substitution of some functional groups' inactive sites can be efficient, but even though this is a good method, focusing on molecular scaffolds to achieve better bioactive compounds is much more efficient [232]. Generally, a ‘scaffold’ is defined as a molecular core attached to functional groups [233–235]. Molecular scaffolds have been investigated by several studies. As an example, Hardcastle *et al*. [236] have investigated isoindolinone as a scaffold to improve MDM2 inhibitor properties. Their results showed that isoindolinone is less efficient than the active compounds, but its structure can be edited to change their substitution and create a new location for the junction of MDM2. Thus, it is possible to create various junctions by using ducking calculations and investigating their properties using computational methods.

Interestingly, it is also possible to investigate these inhibitors and their binding affinity to the target from the point of energetic view relying on the binding free energy composed of both enthalpic and entropic contributions. In fact, we can choose a compound that is energetically more stable by using thermodynamic functions Δ*H* and Δ*G*. Notably, owing to the binding of functional groups to an isoindolinone complex as well as binding of the inhibitor to the target, entropy decreases and we know that a decrease in entropy leads to a decrease in system stability. In these inhibitors, enthalpy–entropy compensation will be used to investigate the interplay between entropy and enthalpy to achieve high affinity of inhibitor and target binding and driving forces of binding of the inhibitor to the target [237].

However, to achieve more efficient inhibitors, we should choose the best scaffold. Towards this goal, one way is to define a feature named TS (target selectivity), which is the difference between p*K*_iA_ and p*K*_iB_ (p*K*_iA_ and p*K*_iB_ are the logarithms of the potential value of target compounds A and B, respectively) [238]. A graph is the best tool to understand scaffold selectivity better. Imagine a graph of all targets in a proteasome–ubiquitin system, which are nodes of this graph. Edges are drawn between the nodes if they have at least five common compounds. Notably, choosing number 5 controls network noise and ensures the reliability of selectivity profiling, as Hu *et al*. [238] indicated. Calculating the TS feature for all targets and all scaffolds in bioactive compounds against these targets showed the best scaffold with the highest TS (between −3 and 3). Interestingly, in a community of targets, a target that has the highest TS for a scaffold can give us the most efficient inhibitor. As shown in a graph, each target is bonded to several other targets. By comparing the common compounds in these targets, we can identify the compound to which the highest TS is related. Identification of these compounds can help us to achieve a scaffold with the highest TS [238]. To change the TS, the p*K*_i_ of the scaffold should be changed.

In order to efficiently change the p*K*_i_, we should know the activity cliffs. Activity cliffs are defined as a pair or groups of similar compounds that are extremely different in potency. These cliffs have been categorized in several groups as ‘chirality cliffs’, ‘topology cliffs’, ‘R-group cliffs’, ‘scaffold cliffs’ and ‘scaffold/topology cliffs' [239]. Among these cliffs, the most important for us are scaffold cliffs. Notably, in these cliffs, the difference is the place of the same substitution [240]. What is important is that scaffold cliffs have different potential, so exchanging them with each other could change the TS value. Of note, according to a synergistic effect, the combination of scaffold cliffs and topology cliffs (cliffs that are different in the position of the same set of substituents in a conserved scaffold) as scaffold/topology cliffs has higher potential than the summation of each individual potential [239]. Therefore, utilization of these cliffs can be more efficient.

However, there are several ways to produce new scaffolds that have the same or different bioactivity from the previous ones experimentally. Generally, to achieve this goal, we can use different procedures like ring-opening, contraction ring, and expansion ring [241]. Particularly, two ways exist to synthesize a scaffold by making a change in it. First, the biology-oriented synthetic (BOS) method is used to produce structurally novel molecules. In this procedure, a scaffold becomes reduced in a stepwise process to convert to an intermediate scaffold used to produce a new scaffold. To this end, both chemical and biological (for example, a gene transferring technique) limitations are common. Biologically, the scaffolds synthesized in this way have the same bioactivity compared to the primary scaffold [241]. The second procedure is fragment biased compound design (FBCD), in which several fragments are used to form a scaffold by conjugating to each other [242]. Conjugation of these fragments to each other occurs in different regions as follows [241]:

(1) by a linear connection,
(2) by an edge (the final scaffold is called fused edge on edge scaffold),
(3) by one atom (the final scaffold is named Spiro scaffold),
(4) by two atoms and one bridge (the final scaffold is called bridge bicyclist scaffold), and
(5) by an intermediate compound (the final scaffold is called bridge bipodal).

Notably, these differences in fragment connections result in different bioactivities. Interestingly, we can use these procedures to connect several scaffolds to produce a new scaffold. Note that properties of a new scaffold such as its bioactivity might be similar to primary scaffolds or synergism. In fact, its bioactivity might be more than each primary scaffold. However, what is notable is that compounds sometimes rotate when they are binding to another compound. This phenomenon leads to the formation of regio-isomeric scaffolds, which are different in their bioactivities [241]. Anyway, exploring scaffolds systematically using a structure-based method has the potential to open new sights in drug discovery for further developments, especially in the field of UPS.

## 12. Conclusion

Despite the passing of several years since the discovery of UPS, much research is still needed to find an efficient treatment for cancer. The success of Bortezomib as an inhibitor of this system increased hope for continuing such research. However, lately, it has been shown that using inhibitors to target the ubiquitination process is more effective. Thus, some inhibitors for E_1_, E_2_ and E_3_ enzymes have been presented here, of which only the E3 enzyme inhibitors were clinically successful since the inhibitors of E_1_ and E_2_ enzymes had poor pharmacokinetic properties. As for the various E_3_ ligases in the cell processes, targeting them for cancer therapy is complex and, therefore, requires more investigation. However, there are several ways exist to improve the bioactivity of the inhibitors, which are available now. One of these is to investigate inhibitor scaffolds to increase their potential. However, more research should be done to find the most useful way, at the lowest cost, to increase anti-cancer translation.

## Data accessibility

The authors confirm that the data supporting the findings of this study are available within the article [and/or] its electronic supplementary material.
